# Detection of Colistin Resistance in Escherichia coli by Use of the MALDI Biotyper Sirius Mass Spectrometry System

**DOI:** 10.1128/JCM.01427-19

**Published:** 2019-11-22

**Authors:** R. Christopher D. Furniss, Laurent Dortet, William Bolland, Oliver Drews, Katrin Sparbier, Rémy A. Bonnin, Alain Filloux, Markus Kostrzewa, Despoina A. I. Mavridou, Gerald Larrouy-Maumus

**Affiliations:** a MRC Centre for Molecular Bacteriology and Infection, Department of Life Sciences, Faculty of Natural Sciences, Imperial College London, London, United Kingdom; b Department of Bacteriology-Hygiene, Bicêtre Hospital, Assistance Publique-Hôpitaux de Paris, Le Kremlin-Bicêtre, France; c EA7361, LabEx Lermit, Faculty of Medicine, Université Paris-Sud, Le Kremlin-Bicêtre, France; d French National Reference Centre for Antibiotic Resistance, Le Kremlin-Bicêtre, France; e Bruker Daltonik GmbH, Bremen, Germany; Medical College of Wisconsin

**Keywords:** diagnostics, Gram-negative bacteria, lipid A, mass spectrometry, polymyxins

## Abstract

Polymyxin antibiotics are a last-line treatment for multidrug-resistant Gram-negative bacteria. However, the emergence of colistin resistance, including the spread of mobile *mcr* genes, necessitates the development of improved diagnostics for the detection of colistin-resistant organisms in hospital settings. The recently developed MALDIxin test enables detection of colistin resistance by matrix-assisted laser desorption ionization–time of flight mass spectrometry (MALDI-TOF MS) in less than 15 min but is not optimized for the mass spectrometers commonly found in clinical microbiology laboratories. In this study, we adapted the MALDIxin test for the MALDI Biotyper Sirius MALDI-TOF MS system (Bruker Daltonics). We optimized the sample preparation protocol by using a set of 6 mobile colistin resistance (MCR) protein-expressing Escherichia coli clones and validated the assay with a collection of 40 E. coli clinical isolates, including 19 confirmed MCR protein producers, 12 colistin-resistant isolates that tested negative for commonly encountered *mcr* genes (i.e., likely chromosomally resistant isolates), and 9 polymyxin-susceptible isolates. We calculated polymyxin resistance ratio (PRR) values from the acquired spectra; PRR values of 0, indicating polymyxin susceptibility, were obtained for all colistin-susceptible E. coli isolates, whereas positive PRR values, indicating resistance to polymyxins, were obtained for all resistant strains, independent of the genetic basis of resistance. Thus, we report a preliminary feasibility study showing that an optimized version of the MALDIxin test adapted for the routine MALDI Biotyper Sirius system provides an unbiased, fast, reliable, cost-effective, and high-throughput way of detecting colistin resistance in clinical E. coli isolates.

## Abstract (precis)

Polymyxin antibiotics are a last-line treatment for multidrug-resistant Gram-negative bacteria. However, the emergence of colistin resistance, including the spread of mobile *mcr* genes, necessitates the development of improved diagnostics for the detection of colistin-resistant organisms in hospital settings.

## INTRODUCTION

Antibiotic resistance is an issue of global importance and one of the defining public health concerns of our time (1). The limited pipeline of novel antimicrobials and the spread of multidrug-resistant (MDR) organisms have increased our reliance on a few last-line antibiotics for the treatment of MDR Gram-negative bacteria. Chief among these last-resort agents are the polymyxin antibiotics, polymyxin B and colistin (2, 3).

In Gram-negative bacteria like Escherichia coli, polymyxin resistance mostly occurs as a consequence of lipopolysaccharide (LPS) modifications, in the form of addition of the cationic groups phosphoethanolamine (pETN) and/or 4-amino-l-arabinose (l-Ara4N) to the lipid A portion of LPS (4, 5). These lipid A modifications often arise due to alterations to the PmrAB and PhoPQ two-component systems, mutations to MgrB, a negative regulator pf PhoP and PhoQ, or the activity of plasmid-borne pETN transferases called mobile colistin resistance (MCR) enzymes (6). The first MCR enzyme, MCR-1, was reported in 2016 (7), and this discovery was followed by the rapid identification of other mobile polymyxin resistance genes. To date, another eight MCR proteins have been described; these enzymes cluster into four main groups, i.e., MCR-1-like (MCR-1, 2, and 6), MCR-3-like (MCR-3, 7, 8, and 9), MCR-4-like (MCR-4), and MCR-5-like (MCR-5) (8–11).

Detection of colistin resistance currently relies on MIC determinations using broth microdilution (BMD), a slow process that, despite being the gold standard for polymyxin susceptibility testing, has been subject to reliability and standardization problems (6, 12). Additionally, routine detection of colistin resistance by conventional methods such as PCR-based testing is challenging due to the wide range of chromosomal mutations that can give rise to colistin resistance (6) and the low sequence identity of the *mcr* genes (using *mcr-1* as a reference, identity is as follows: *mcr-2*, 77.6%; *mcr-3*, 49.2%; *mcr-4*, 46.8%; *mcr-5*, 50. 5%; *mcr-6*, 78.3%; *mcr-7*, 49.9%; *mcr-8*, 47.8%; *mcr-9*, 57.69%). This means that PCR-based detection methods are insensitive to all but the best-characterized chromosomal mutations, as well as to the emergence of new *mcr* genes. Therefore, there is an urgent need to develop a fast, robust, and high-throughput assay that is accessible to all diagnostic microbiology laboratories and uses an unbiased approach to detect colistin resistance arising from both known and novel chromosomal mutations or MCR proteins.

Recently, we developed the MALDIxin test, a diagnostic tool based on matrix-assisted laser desorption ionization–time of flight mass spectrometry (MALDI-TOF MS) that can be used to detect colistin resistance using intact bacteria in less than 15 minutes (13, 14). Although it is fast and effective, this test was not optimized for routine use in diagnostic microbiology laboratories, with the main limitation being that it was not developed for the MALDI-TOF mass spectrometers that are widely used for bacterial identification in such settings. More specifically, our previous studies were performed on a research instrument operating in the high-resolution reflector mode, while MALDI-TOF MS systems in clinical microbiology laboratories employ lower-resolution measurements in linear mode. Here, we report a preliminary feasibility study showing that an optimized version of the MALDIxin test designed for the low-resolution linear mode employed by the MALDI Biotyper Sirius system (Bruker Daltonics) accurately identifies colistin resistance in clinical E. coli isolates, irrespective of its genetic basis, by detecting the addition of both pETN and l-Ara4N moieties to lipid A.

## MATERIALS AND METHODS

### Bacterial strains.

For the construction of MCR protein-producing E. coli clones (Table 1), *mcr* variants were cloned into pDM1 (GenBank accession no. MN128719), an isopropyl-β-d-1-thiogalactopyranoside (IPTG)-inducible derivative of pACYC184; protein expression from this vector is induced only after addition of IPTG to the culture medium. The SacI and XmaI sites of the vector were used for *mcr-1*, *mcr-2*, *mcr-4*, *mcr-5*, and *mcr-8*, while the NdeI and XmaI sites were used for *mcr-3*. A collection of 40 E. coli clinical isolates (Table 1), including 19 confirmed MCR protein producers, 12 colistin-resistant isolates that tested negative for commonly encountered *mcr* genes, and 9 colistin-susceptible isolates, was used for blinded validation of the MALDIxin test.

**TABLE 1:** PRR values for MCR protein-producing E. coli clones and colistin-resistant and colistin-susceptible clinical E. coli strains used in this study^*a*^

Strain	Colistin MIC (mg/liter)	Resistance mechanism	Additional β-lactamase gene(s)	PRR	PRR^1,919^	PRR^1,927^
MCR protein-producing E. coli clones						
MC1000 pDM1-*mcr-1*	4	*mcr-1*		6.63 ± 0.68	6.63 ± 0.68	0.00 ± 0.00
MC1000 pDM1-*mcr-2*	4	*mcr-2*		4.80 ± 0.72	4.80 ± 0.72	0.00 ± 0.00
MC1000 pDM1-*mcr-3*	4	*mcr-3*		4.54 ± 0.15	4.54 ± 0.15	0.00 ± 0.00
MC1000 pDM1-*mcr-4*	4	*mcr-4*		4.47 ± 0.78	4.47 ± 0.78	0.00 ± 0.00
MC1000 pDM1-*mcr-5*	4	*mcr-5*		4.00 ± 1.29	4.00 ± 1.29	0.00 ± 0.00
MC1000 pDM1-*mcr-8*	4	*mcr-8*		3.36 ± 1.44	3.36 ± 1.44	0.00 ± 0.00
MC1000 pDM1	0.5			0.00 ± 0.00	0.00 ± 0.00	0.00 ± 0.00
Colistin-resistant strains harboring *mcr* genes						
CNR 20140385	4	*mcr-1*	OXA-48	0.91 ± 0.18	0.91 ± 0.18	0.00 ± 0.00
S08-056	4	*mcr-1*	OXA-48	1.86 ± 0.28	1.86 ± 0.28	0.00 ± 0.00
CNR 117 G7	4	*mcr-1*	NDM-1	1.70 ± 0.68	1.70 ± 0.68	0.00 ± 0.00
CNR 1745	4	*mcr-1*	SHV-12	1.65 ± 0.02	1.65 ± 0.02	0.00 ± 0.00
CNR 1604	4	*mcr-1*	CTX-M-15	1.98 ± 0.30	1.98 ± 0.30	0.00 ± 0.00
CNR 1790	4	*mcr-1*	TEM-15	1.37 ± 0.05	1.37 ± 0.05	0.00 ± 0.00
CNR 1859	4	*mcr-1*	CTX-M-15, SHV-12, TEM-1	2.95 ± 0.10	2.95 ± 0.10	0.00 ± 0.00
CNR 1886	4	*mcr-1*	CTX-M-1, TEM-1	1.05 ± 0.11	1.05 ± 0.11	0.00 ± 0.00
4222	4	*mcr-1*	CTX-M-2	1.75 ± 0.42	1.75 ± 0.42	0.00 ± 0.00
4070	4	*mcr-1*	TEM-1B	0.98 ± 0.03	0.98 ± 0.03	0.00 ± 0.00
979	4	*mcr-1*	CTX-M-2	1.75 ± 0.26	1.75 ± 0.26	0.00 ± 0.00
1724	4	*mcr-1*		1.28 ± 1.21	1.28 ± 1.21	0.00 ± 0.00
CNR 164 A5	4	*mcr-1*		1.56 ± 0.44	1.56 ± 0.44	0.00 ± 0.00
1670	4	*mcr-1.5*	CTX-M-2	1.21 ± 0.32	1.21 ± 0.32	0.00 ± 0.00
6383	4	*mcr-1.5*	TEM-1B	1.75 ± 0.08	1.75 ± 0.08	0.00 ± 0.00
R12 F5	4	*mcr-2*		0.66 ± 0.08	0.66 ± 0.08	0.00 ± 0.00
37922	4	*mcr-3.2*	CTX-M-55	1.52 ± 0.18	1.52 ± 0.18	0.00 ± 0.00
1144230	4	*mcr-5*	CMY-2	1.09 ± 0.20	1.09 ± 0.20	0.00 ± 0.00
J53 pMCR-8 (Kpn)	4	*mcr-8*		0.25 ± 0.03	0.25 ± 0.03	0.00 ± 0.00
Colistin-resistant strains negative for *mcr-1* to *mcr-5*						
CNR 111 J7	8	PmrB (D14N, S71C, V83A)		1.20 ± 1.00	0.40 ± 0.50	0.80 ± 0.50
CNR 20160039	4	Unknown	Penicillinase	1.40 ± 0.20	0.50 ± 0.10	0.90 ± 0.10
CNR 20160235	4	MgrB (V8A)		0.90 ± 0.31	0.00 ± 0.00	0.90 ± 0.31
CNR 1728	8	PmrB (G160E)		1.20 ± 0.30	0.50 ± 0.10	0.70 ± 0.20
CNR 187 G3	4	Unknown	NDM-5	0.10 ± 0.00	0.00 ± 0.00	0.10 ± 0.00
CNR 189 E5	4	Unknown	NDM-5	0.10 ± 0.00	0.00 ± 0.00	0.10 ± 0.00
CNR 169 D6	4	Unknown	OXA-48	0.20 ± 0.00	0.00 ± 0.00	0.20 ± 0.00
CNR 165 J9	4	Unknown	ESBL^*b*^	0.90 ± 0.00	0.00 ± 0.00	0.90 ± 0.00
CNR 196 G2	4	Unknown	ESBL	1.01 ± 0.35	1.01 ± 0.35	0.00 ± 0.00
CNR 169 F2	8	Unknown		0.60 ± 0.10	0.00 ± 0.00	0.60 ± 0.10
CNR 198 E2	8	Unknown		0.40 ± 0.10	0.00 ± 0.00	0.40 ± 0.10
CNR 181 D5	16	Unknown	VIM-1	0.70 ± 0.00	0.6 ± 0.00	0.10 ± 0.00
Colistin-susceptible strains						
J53	0.5			0.00 ± 0.00	0.00 ± 0.00	0.00 ± 0.00
1608071881	0.25			0.00 ± 0.00	0.00 ± 0.00	0.00 ± 0.00
1608075385	0.12		Penicillinase	0.00 ± 0.00	0.00 ± 0.00	0.00 ± 0.00
1608078105	0.25		Penicillinase	0.00 ± 0.00	0.00 ± 0.00	0.00 ± 0.00
2H6	0.25		CTX-M-15	0.00 ± 0.00	0.00 ± 0.00	0.00 ± 0.00
2E10	0.25		CTX-M-14	0.00 ± 0.00	0.00 ± 0.00	0.00 ± 0.00
1A6	0.25		NDM-4, CTX-M-15, OXA-1	0.00 ± 0.00	0.00 ± 0.00	0.00 ± 0.00
1C2	0.5		VIM-1	0.00 ± 0.00	0.00 ± 0.00	0.00 ± 0.00
2A1	0.25		OXA-48, CTX-M-15	0.00 ± 0.00	0.00 ± 0.00	0.00 ± 0.00

a Values shown are the mean ± standard deviation of three independent experiments. Where known, the genetic basis of resistance is indicated (resistance mechanism). PRR values were calculated by summing the intensities of the lipid A peaks attributable to the addition of pETN (*m/z* 1,919.2) and l-Ara4N (*m/z* 1,927.2) and dividing this number by the intensity of the peak corresponding to native lipid A (*m/z* 1,796.2) (PRR = [*m/z* 1,919.2 intensity +  *m/z* 1,927.2 intensity]/*m/z* 1,796.2 intensity). PRR^1,919^ and PRR^1,927^ indicate the contributions of specific lipid A modifications (pETN and/or l-Ara4N) to the overall PRR value. PRR^1,919^ and PRR^1,927^ were calculated by dividing the intensity of the peak at the appropriate *m/z* value (*m/z* 1,919.2 and *m/z* 1,927.2 for pETN and l-Ara4N addition, respectively) by the intensity of the peak corresponding to native lipid A (*m/z* 1,796.2).

b ESBL, extended-spectrum β-lactamase.

### Genotype determination.

PCR-based amplification and DNA sequencing were used to determine the genotypes of all tested clinical isolates (Table 1). Identification of commonly encountered *mcr* genes (*mcr-1*, *mcr-2*, *mcr-3*, *mcr-4*, and *mcr-5*) was performed by multiplex PCR, as described previously (15), and β-lactamase genes were identified using in-house multiplex PCR protocols. Colistin-resistant isolates that were negative for the tested *mcr* genes (*mcr-1*, *mcr-2*, *mcr-3*, *mcr-4*, and *mcr-5*) by multiplex PCR were considered to be resistant most likely due to a chromosomally encoded mechanism.

### Susceptibility testing.

Colistin MICs for clinical isolates were manually determined using BMD, according to the Clinical and Laboratory Standards Institute (CLSI) and European Committee on Antimicrobial Susceptibility Testing (EUCAST) guidelines. Therefore, cation-adjusted Mueller-Hinton broth was used in conjunction with plain polystyrene laboratory consumables and the sulfate salt of colistin. No additives were used at any stage of the testing process. For the laboratory E. coli clones, which were used only for protocol optimization, 0.5 mM IPTG was added to the BMD growth medium to induce expression of the MCR enzymes. The colistin MICs for all tested strains were determined three times and were found to be identical in each repeat. Results were interpreted using EUCAST breakpoints (16).

### Optimized MALDIxin test for the MALDI Biotyper Sirius system.

A 10-μl inoculation loop of bacteria, grown on Mueller-Hinton agar for 18 to 24 h, was resuspended in 200 μl of water. Mild acid hydrolysis was performed on 100 μl of this suspension, by adding 100 μl of 2% (vol/vol) acetic acid and incubating the mixture at 98°C for 5 min. Hydrolyzed cells were centrifuged at 17,000 × *g* for 2 min, the supernatant was discarded, and the pellet was resuspended in ultrapure water to a McFarland standard of 10. A 0.4-μl aliquot of this suspension was loaded onto the target and immediately overlaid with 1.2 μl of a matrix consisting of a 9:1 mixture of 2,5-dihydroxybenzoic acid and 2-hydroxy-5-methoxybenzoic acid (super-DHB) (Sigma-Aldrich) dissolved in 90:10 (vol/vol) chloroform/methanol to a final concentration of 10 mg/ml. The bacterial suspension and matrix were mixed directly on the target by pipetting, and the mixture was dried gently under a stream of air for less than 1 min. MALDI-TOF MS analysis was performed with a MALDI Biotyper Sirius system (Bruker Daltonics), using the newly introduced linear negative-ion mode.

### Data analysis.

Manual peak picking at masses relevant to colistin resistance was performed on the mass spectra obtained, and the corresponding signal intensities at the defined masses were determined. Peaks were considered only if their signal/noise ratio was at least 5. The sum of the intensities of the lipid A peaks attributed to the addition of pETN (*m/z* 1,919.2) or l-Ara4N (*m/z* 1,927.2) was divided by the intensity of the peak corresponding to native lipid A (*m/z* 1,796.2). The resulting value is termed the polymyxin resistance ratio (PRR). A PRR of 0 indicates colistin susceptibility, while a positive value indicates colistin resistance. PRR^1,919^ values were determined by dividing the intensity of the peak at *m/z* 1,919 alone by the native lipid A peak, and PRR^1,927^ values were determined by dividing the intensity of the peak at *m/z* 1,927 alone by the native lipid A peak. All mass spectra were generated and analyzed in technical triplicate (i.e., measurements of each sample were repeated three times) and biological triplicate (i.e., the entire experiment was repeated on 3 separate days, using separately grown bacteria and separate materials). For all clinical isolates, operators were blinded during data collection and analysis.

### Data availability.

Sequence data were deposited in GenBank under accession no. MN128719.

## RESULTS

**FIG 1:**
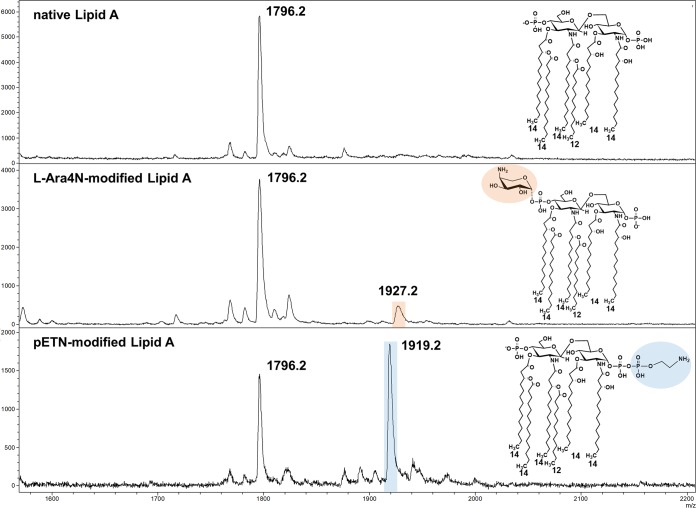
Representative mass spectra of native and modified E. coli lipid A, acquired using the linear negative-ion mode of a MALDI Biotyper Sirius system (Bruker Daltonics). Native E. coli lipid A is detected as one major peak at *m/z* 1,796.2 (top). Lipid A from colistin-resistant E. coli isolates carrying chromosomal mutations is modified with l-Ara4N, which is detected as an additional peak at *m/z* 1,927.2 (highlighted in orange) (middle), and/or pETN, which is detected as an additional peak at *m/z* 1,919.2 (highlighted in blue) (bottom). Lipid A from strains exhibiting MCR protein-mediated resistance to colistin is modified only with pETN (bottom); the spectrum shown in the bottom panel is typical of an *mcr*-carrying isolate. Insets show the corresponding structures of native and modified lipid A, with the l-Ara4N and pETN modifications highlighted.

To allow the use of the MALDIxin test on the MALDI Biotyper Sirius system, it was necessary to optimize the sample preparation protocol. This optimization was carried out using a panel of 6 isogenic E. coli clones expressing representative members of each of the major MCR groups (MCR-1, 2, 3, 4, 5, and 8) and an E. coli clone carrying the expression vector (pDM1) alone (Table 1). For the E. coli clone carrying only the expression vector, the negative-ion mass spectrum scanned between *m/z* 1,600 and *m/z* 2,200 was dominated by a set of peaks assigned to bisphosphorylated hexa-acyl lipid A. The major peak at *m/z* 1,796.2 corresponds to hexa-acyl diphosphoryl lipid A containing four 3-OH-C14:0 acyl groups, one C14:0 acyl group, and one C12:0 acyl group, referred to as native lipid A (Fig. 1, top). For E. coli clones expressing MCR enzymes, the addition of pETN to the 1-phosphate of native lipid A leads to an additional peak (*m/z* 1,919.2) that is shifted by *m/z* +123, compared to the mass of the major peak at *m/z* 1,796.2 (Fig. 1, bottom). The sample optimization process aimed to achieve a signal/noise ratio of >10 for the peaks at *m/z* 1,796.2 and *m/z* 1,919.2. For this purpose, the sample preparation procedure was divided into three steps, (i) acid hydrolysis, (ii) sample washing, and (iii) sample resuspension prior to MALDI-TOF MS analysis. Parameters such as the acetic acid concentration, the time of hydrolysis, the sample washing procedure after acid hydrolysis, and the sample density after resuspension were adjusted accordingly. The final optimized protocol is detailed in Materials and Methods.

The optimized version of the MALDIxin test was blindly validated using a panel of 40 E. coli clinical isolates (Table 1), including 19 confirmed MCR protein producers, 12 colistin-resistant isolates that tested negative for *mcr-1* to *mcr-5* genes and were most likely resistant due to chromosomally encoded mechanisms, and 9 colistin-susceptible isolates. For all colistin-susceptible E. coli clinical isolates in this panel, a single peak at *m/z* 1,796.2 was detected, confirming that the lipid A in these strains was unmodified. For all MCR protein producers, both the native lipid A peak and the additional pETN peak at *m/z* 1,919.2 were observed, independent of the amino acid sequence of the MCR protein conferring colistin resistance. For all but 1 of the colistin-resistant isolates that were not found to carry a commonly encountered *mcr* gene by multiplex PCR (15) and thus were likely to harbor chromosomal mutations leading to colistin resistance, we were able to detect a peak at *m/z* 1,927.2 in addition to the native lipid A peak. This signal corresponds to the addition of l-Ara4N to the 4ʹ-phosphate of lipid A, resulting in an increase of *m/z* +131, compared to the native lipid A peak (Fig. 1, middle). For several of these isolates, peaks at both *m/z* 1,919.2 and *m/z* 1,927.2 were observed, suggesting that these organisms possessed lipid A species modified with both pETN and l-Ara4N (Table 1). Finally, for 1 of these isolates (CNR 196 G2), we detected only a peak at *m/z* 1,919.2, indicating that its lipid A was modified solely by pETN (Table 1). Using these spectra, PRR values for all strains were calculated. Importantly, all susceptible E. coli strains were found to have a PRR value of 0, while all colistin-resistant isolates, independent of their identified lipid A modifications, had positive PRR values (Table 1). While the PRR value clearly indicates whether an isolate is resistant or susceptible to colistin, the contribution of each lipid A modification (Fig. 1) to the overall PRR value, and thus colistin resistance, can be further assessed by calculating PRR^1,919^ (pETN) and PRR^1,927^ (l-Ara4N) values (Table 1). Although chromosomal resistance cannot be excluded, isolates for which the PRR^1,919^ value is equal to the PRR value are likely MCR protein producers. In contrast, isolates for which the PRR value is equal to the PRR^1,927^ value, or the sum of the PRR^1,919^ and PRR^1,927^ values, are almost certainly resistant by a chromosomally encoded mechanism, such as a *pmrB* mutation.

## DISCUSSION

The work presented here broadens the applicability of our previously developed MALDIxin test (13) and represents an unbiased, fast, cost-effective, and high-throughput method to detect colistin resistance in E. coli by directly assessing the biochemical cause of resistance, i.e., the modification of lipid A. Therefore, unlike PCR-based testing, this method can reliably identify clinical isolates harboring chromosomal mutations, *mcr* genes, and novel colistin resistance determinants, such as emerging MCR members, regardless of the genetic basis of resistance. Indeed, by determining the lipid A modification(s) responsible for colistin resistance through the calculation of PRR^1,919^ and PRR^1,927^ values, potential MCR protein producers (i.e., organisms for which the PRR value is attributable solely to the addition of pETN to lipid A) can be quickly identified for future in-depth characterization, using approaches such as multiplex PCR or whole-genome sequencing.

For this analysis, we used the recently released MALDI Biotyper Sirius mass spectrometer. This system differs from previous Biotyper systems in that it can operate in both positive- and negative-ion modes. Analytes that are acidic in nature, such as those containing phosphate or carboxylate groups, are more efficiently ionized by the generation of anions (17). Therefore, detection of lipid A, which contains both long-chain fatty acid and phosphate groups (at carbons 1 and 4′), is superior when anions are generated using the negative-ion mode. The newly introduced negative-ion mode of the MALDI Biotyper Sirius allows efficient detection of both native lipid A and its modified forms. Although the MALDI Biotyper Sirius is the optimal mass spectrometer for the assay as described here, lipid A can be detected using any MALDI-TOF MS mass spectrometer that supports negative-ion mode. In addition to the newly introduced negative-ion mode, the MALDIxin test uses a super-DHB MALDI matrix, as opposed to the α-cyano-4-hydroxycinnamic acid (HCCA) matrix routinely used for bacterial identification by MALDI-TOF MS. While both super-DHB and HCCA are traditional organic matrices, super-DHB is a binary mixture of two benzoic acid derivatives. Mixed matrices such as super-DHB offer improved yields and signal/noise ratios for analyte ions by altering the cocrystallization of the analyte and matrix components (18). Together, these two advances allow the MALDI Biotyper Sirius to be used for both bacterial identification and colistin resistance determination, through detection of native or modified lipid A from whole bacterial colonies.

The modification of lipid A is a common mechanism of colistin resistance in organisms beyond E. coli. Because the structure of lipid A from a range of bacterial species (including Klebsiella pneumonia, *Shigella* spp., and Pseudomonas aeruginosa) can be determined by MALDI-TOF MS (19), this technique provides a broadly applicable basis for the development of new diagnostics for many species of Gram-negative bacteria. Indeed, the lipid A of *Salmonella* spp., which have been reported to carry MCR enzymes (20), is similar to that of E. coli and can be detected, using the negative-ion mode of the MALDI Biotyper Sirius system, as a peak at *m/z* 1,796.2 (data not shown). Thus, it is likely that a similar *m/z* +123 addition to the native lipid A peak would be observed in colistin-resistant isolates of this organism. Similarly, lipid A from Acinetobacter baumannii can be detected directly by using MALDI-TOF MS; colistin resistance in this organism, primarily resulting from the overexpression of the chromosomally encoded pETN transferase PmrC, can be detected as a *m/z* +123 addition to the peak corresponding to native bisphosphorylated hepta-acyl lipid A (13). These observations suggest that the optimized version of the MALDIxin test presented here will have broad utility in detecting colistin resistance in a range of Gram-negative bacteria.

The diagnostic assay described in this study will initially be made available to users of the MALDI Biotyper Sirius, along with full application support, for research use only (RUO) validation studies. This will be followed by the transformation of an already existing, RUO, web-based, automated algorithm (Bruker Daltonics) into a new MALDI Biotyper software module. Dedicated MALDIxin consumables (e.g., preportioned purified matrix and calibration standards) will also be developed, to enable simplified and standardized performance of the assay. Successful deployment of the new software module, in conjunction with MALDIxin-specific laboratory consumables, will allow subsequent introduction of *in vitro* diagnostic consumables and software, following further clinical and analytical studies. These steps will ultimately bring the MALDIxin test into clinical laboratories in the near future.

Overall, this study represents a major step toward the routine application of MALDI-TOF MS-based detection of colistin resistance and lays the foundations for a rapid diagnostic test for colistin resistance that will be readily accessible to most clinical microbiology laboratories. Adoption of the MALDI Biotyper Sirius system, and the subsequent introduction of the MALDIxin test, will facilitate improved treatment of patients with challenging MDR Gram-negative infections.
